# Nurses’ experiences of the family’s role in end-of-life care

**DOI:** 10.4102/hsag.v29i0.2565

**Published:** 2024-05-23

**Authors:** Lesley A. Paterson, Jeanette E. Maritz

**Affiliations:** 1 Department of Health Studies, University of South Africa, Pretoria, South Africa

**Keywords:** end-of-life care, nursing, patients’ family, palliative care, end-of-life training

## Abstract

**Background:**

Family units can be deeply affected and require nurses’ attention and care when their loved ones reach the end-of-life stage. However, the role of the family in end-of-life nursing is under-researched in a tertiary hospital setting.

**Aim:**

This study aims to explore and describe nurses’ lived experiences of the family’s role in end-of-life care at a tertiary hospital in the Western Cape.

**Setting:**

The study took place in a tertiary hospital in the Western Cape.

**Methods:**

A qualitative hermeneutic design with a phenomenological approach was used. Ten professional nurses were interviewed in person, and two focus groups comprised enrolled and auxiliary nurses (11) with a minimum of 6 months of experience in end-of-life care. Data analysis was conducted by categorising qualitative information into codes and themes, following Creswell’s outlined methodology.

**Results:**

Three themes emerged: emotional challenges associated with families of end-of-life patients, strategies that assist families and impediments to providing care. Participants experienced challenges managing strong emotions expressed by families and their own, but provided care regardless. Strategies that have been found to be helpful to families include communication, access, and involving them in patient care. Participants experienced the need for emotional support and specialised end-of-life training.

**Conclusion:**

Participants strive to provide understanding and support to families despite challenges, but emotional and end-of-life training resources are required to equip nurses to address patients’ and families’ needs competently.

**Contribution:**

This study adds the nurses’ experience and understanding of the family’s role in providing end-of-life care in a tertiary hospital.

## Introduction

End-of-life (EOL) care, or palliative care, provides essential support to individuals at the end of their lives or facing terminal illnesses. This support covers medical, psychological and spiritual aspects, aiming to improve the quality of life by alleviating suffering and addressing comprehensive needs. The approach is holistic, focusing on symptom management and supporting the patient’s family and loved ones to ensure comfort, dignity and respect in accordance with the patient’s wishes (Akdeniz, Yardımcı & Kavukcu 2021:1–9; Rosa, Ferrell & Mazanec 2021:131).

The EOL process involves complex medical, emotional and ethical considerations affecting patients, their families and healthcare providers. Despite the World Health Organization’s (2020)call for universal palliative care access, the practical delivery often relies on nurses, who encounter many challenges in healthcare settings. Families, crucial in this context, face significant distress associated with the imminent loss of a loved one, highlighting a ‘reciprocity of suffering’ (De Beer & Brysiewicz 2019:19–23; Steele & Davies 2015:51–72). This reciprocal dynamic underscores the complex interplay of emotions and experiences within the family unit, where the distress of one member resonates throughout the family, emphasising the importance of holistic palliative care that extends beyond the patient to address the needs and suffering of the entire family network.

However, the literature often overlooks families’ essential role and needs in EOL care, especially in specific contexts like South African tertiary hospitals. This oversight signals a need for further investigation into the family’s role in EOL care within these environments. The significance of family in navigating the complexities of illness, injury or EOL situations is paramount across all healthcare settings, impacting family dynamics (De Beer & Brysiewicz 2019:19).

In the context of South Africa, shaped by apartheid-induced disparities and a pronounced private–public healthcare divide, these challenges are magnified. The system, skewed towards the affluent in the private sector, leaves most patients reliant on an overstretched public sector. The South African public healthcare sector faces significant challenges exacerbated by the coronavirus disease 2019 (COVID-19) pandemic, including a severe shortage of healthcare professionals, extended working hours and a lack of essential resources. These issues are compounded by systemic problems such as poor leadership, inadequate resource allocation and persistent inequalities in access to healthcare, particularly for those in densely populated areas. The overstretched public sector needs help managing the increased pressure and extreme working conditions, further straining an already burdened system (Abrahams, Thani & Khan 2022:63–66). This backdrop of inequity and efforts to shift towards comprehensive primary healthcare underscores the urgency of exploring EOL care within public hospitals to understand and address the disparities and needs of a broader, more diverse patient population.

Chan et al. (2020:1209–1214) offer insights into nurses’ perceptions and the barriers they face in delivering optimal EOL care in hospitals, highlighting the critical role of healthcare professionals in facilitating family involvement in patient care. This study underscores the importance of addressing communication barriers, enhancing professional training and fostering a supportive care environment as key strategies to improve EOL care outcomes for patients and their families. Barchielli et al. (2023:123), Smaling et al. (2023:5), Bayliss et al. (2023:vi) and Poon, Ang and Ramazanu (2023:219) further explore various strategies, emphasising the importance of caregiver support, cross-cultural acceptability of interventions and the adaptation of care in the digital era.

Therefore, this study aims to explore nurses’ lived experiences concerning the family’s role in EOL care within the public healthcare setting. Focusing on this under-researched area, the research seeks to uncover strategies to enhance EOL care for patients and their families, contributing valuable insights to the discourse on EOL care and informing policy and practice. The guiding research question, ‘What are the lived experiences of nurses regarding the family’s role in end-of-life care?’, positions this investigation to illuminate the complexities surrounding EOL care in a context marked by significant socio-economic and healthcare disparities.

## Aim

This study aimed to explore and describe nurses’ lived experiences of the family’s role in EOL care at a tertiary hospital in the Western Cape.

## Research design and method

### Research design

This study employed a qualitative, hermeneutic phenomenological design to delve into the nuanced and complex nature of the lived experiences under investigation. Hermeneutic phenomenology is particularly suited to exploring the depth of human experiences by interpreting the subjective and interrelated layers of these experiences (Nigar 2020:15 as cited by Leigh-Osroosh 2021). This approach was chosen because it allows for a comprehensive understanding of the ethical, relational and practical dimensions of the phenomenon at hand, aligning with the study’s aim to uncover the intricate realities faced by people (Fuster 2019:210). Furthermore, the evolution of hermeneutic phenomenology from a philosophical grounding to a practical methodology makes it an apt choice for fields such as nursing, where understanding the lived experiences of patients and healthcare professionals can inform and improve practice (Sloan & Bowe 2014:1292). The decision to use this design was driven by its potential to generate rich, detailed descriptions that enhance nurses’ understanding of complex phenomena, thereby facilitating more empathetic and effective care (Creswell & Creswell Báez 2021:5–7).

### Research setting

This study was conducted with nurses working at a tertiary hospital in the Western Cape. The choice was driven by its status as the largest hospital in the Western Cape and the second largest in South Africa, serving a large patient population of over 3.6 million (Western Cape Government). Its comprehensive range of specialty services, academic affiliation and significant bed capacity of 1348 makes it an ideal setting for investigating EOL care. The hospital’s robust referral system and its role as a hub for academic research and clinical training offer a unique opportunity to gain in-depth insights into nurses’ lived experiences with EOL care.

### Population and sample

Male and female nurses over 18 years and under 65 years of age, registered with the South African Nursing Council (SANC) according to the *Nursing Act, Act No. 33 of 2005*, working with terminally ill patients for 6 months or longer, were invited to participate in this study.

The population included professional, enrolled and auxiliary (assistant) nurses who were permanently employed in the hospital’s Internal Medicine Department, a staff complement of 192 nurses working day and night shifts (Table 1).

**TABLE 1:** Nurse population.

Area	Enrolled nursing assistant	Enrolled nurse	Professional nurse
Medical and geriatric ward or units	71	30	46
Oncology wards or units	16	9	20

**Total**	**87**	**39**	**66**

Purposeful sampling was used. Data were collected until data saturation occurred, meaning no new information emerged (Korstjens & Moser 2018:11).

### Data collection

Data were collected from April to October 2018 by researcher LP, who is experienced in specialist nursing and management, where daily practice involves EOL care. Unlike Husserl’s phenomenology, which requires bracketing personal views, hermeneutic phenomenology embraces the researcher’s experiences as part of co-creating reality, enriching data interpretation and authenticity of findings (Arunasalam 2018:np; Sloan & Bowe 2014:1295).

Data were collected using in-depth, face-to-face interviews with professional nurses (*n* = 10) and focus group interviews with enrolled and auxiliary nurses (*n* = 11). With their advanced training and broader scope of practice, professional nurses possess in-depth knowledge and experiences that were best explored through individual interviews, allowing for a focused, detailed examination of their perspectives on EOL care. In contrast, enrolled and auxiliary nurses, who often have more direct, routine interactions with patients but operate under the supervision of professional nurses, might share common experiences that emerge more naturally in the dynamic setting of a focus group. This approach not only respects the hierarchical and functional differences between these groups but also tailors the data collection method to the unique contributions each group can offer to the study (Krueger & Casey 2015:21), ensuring that the insights gathered are both comprehensive and contextually relevant to their specific roles in patient care.

The researcher circulated informational materials and consent forms about the study to the internal, cancer and geriatric wards. These materials were provided to the operational managers, who served as the gatekeepers and then notified the researcher of individuals within their respective wards who expressed interest in participating in the study. Those who met the inclusion criteria and agreed to participate in the study provided their contact details. The researcher then collected these forms, conducted follow-ups through phone calls and arranged the dates and preferred times for interviews.

Piloting interviews and focus groups before the main study helped make questions clearer and group discussions smoother. It allowed for changes to be made based on early feedback, ensuring the final study would gather useful information effectively. Participants were interviewed at the research site in seminar or conference rooms, which allowed for privacy and no noise. Nurses were interviewed during their break periods to avoid impacting service delivery because they were reluctant to relinquish their days off from duty. The interviews were digitally recorded. The interview guideline consisted of an open question with relevant follow-up probes for the in-depth individual interviews and open-ended questions for the focus group interviews (Hennink, Hutter & Bailey 2019:119–148). The grand tour question was: ‘Tell me what it is like for you to care for EOL patients within the ward or unit where you are working at this institution’. The researcher used facilitative communication techniques, including non-verbal cues, clarifications and summaries, to encourage participation and depth in the conversation. These methods ensured engagement and comprehension among participants.

Two focus groups were conducted in seminar or conference rooms during break periods, ensuring a comfortable and convenient setting for participants. The first group of enrolled and auxiliary comprised six participants, and the second group had five participants. All sessions were digitally recorded to capture the discussions accurately. Upon arrival, participants were greeted and encouraged to choose their seating, fostering a welcoming atmosphere. The session began with establishing ground rules and an agenda overview, providing clarity and structure. The same grand tour question initiated the dialogue, guiding the discussion towards the core experiences of enrolled and auxiliary nurses with EOL care. The focus groups were concluded once data saturation was reached, which was indicated by repetitive and similar information. This closure was marked by a collective acknowledgement of the participants’ contributions, emphasising the value of their shared insights into EOL care practices.

### Data analysis

The analytical method in hermeneutic phenomenology does not necessarily follow a single, formalised approach, and the context and the phenomenon itself dictate how data are analysed (Langdridge 2007, as cited by Sloan & Bowe 2014:14). In this study, data analysis was adapted from the steps for qualitative data analysis as indicated by Creswell and Creswell (2023:207–209) and included the following: (1) recorded interviews were transcribed verbatim into text; (2) reading and rereading of transcripts to gain a sense of emerging concepts and general meaning; (3) coding the data by placing text into categories and allocating descriptive labels; (4) clustering related categories into themes; (5) crafting a master table composed of themes, categories and subcategories; and (6) interpreting and describing the phenomenon supported by extracts from interviews (Greening 2019:91).

### Trustworthiness of the study

Trustworthiness refers to confidence in the research process followed and the strategies employed to ensure the quality of a study. A study’s trustworthiness is maintained by preserving its credibility, dependability, confirmability and transferability (Creswell & Creswell Báez 2021).

#### Credibility

In this study, there is alignment between the research question, theory, data collection, analysis and the results, which, according to Stenfors, Kajamaa and Bennett (2020:590), enhances the credibility of the research. The study’s credibility was further improved by investing sufficient time with the participants and ensuring their engagement, as (Creswell & Creswell 2023) suggested. The researcher employed reflexive notes on aspects of the interviews and observations and reflected on her own thoughts and responses (Korstjens & Moser 2018:123).

The researcher coded the data, and the data were also independently coded by an expert in qualitative research. Consensus was reached on the major themes and categories, which gives credibility to the findings (Creswell & Creswell Báez 2021:158–198). Although data were collected in 2018 as part of a doctoral study, the credibility and reliability of the findings were confirmed in October 2023 by a focus group of some of the original professional, enrolled and auxiliary nurses who were involved in the second phase of the research when strategies were formulated.

#### Confirmability and dependability

The researcher collected and analysed the data objectively, as (Creswell & Creswell 2023) indicated. Confirmation of the findings was achieved by creating a clear link between the data and the findings through detailed descriptions and quotes (Stenfors et al. 2020:591). The researcher provided an audit trail comprising transcripts, recordings and field notes.

#### Transferability

Transferability implies that the findings of a study may be transferable or applicable to another separate setting, context, group or research. To improve transferability, a detailed description of the context and method was provided (Stenfors et al. 2020:599). While this study was confined to one tertiary hospital in a single province, the findings could be transferable to other healthcare settings and contribute to the furtherance of EOL care.

### Ethical considerations

Permission for the study was granted by the University of South Africa Ethics Committee (HSHDC/693/2017) and the management and research department of the public hospital where the study was conducted. All participants were informed about the study in writing and explained to them before the interviews and focus groups. Written consent was obtained for participation and recording of the interview (Creswell & Creswell 2023:220). Owing to the topic’s sensitive nature, a psychologist was available for counselling should any participant have required this service during or after the interview process. Participation was voluntary, and participants could withdraw at any point. Participant anonymity was ensured by not linking names or personal information to any data. Data were securely stored on a computer and was password protected. Upon completion of the study, the data will be securely deleted from all digital storage devices. Any physical copies will be shredded, adhering to the Ethics Committee’s specific protocols for data disposal to maintain confidentiality and integrity.

## Results and discussion

The study’s findings are presented and discussed in terms of the related literature.

### Demographic information

The demographic details of the professional nurses interviewed individually are presented in Table 1. The sample’s demographic profile primarily reflected women’s perspectives, as only one male participant existed. The ethnicity of the sample was predominantly *mixed race*, which is representative of the Western Cape, where 49% of the people identify as *mixed race*, while 33% are black people and only 16% are white. However, this is not representative of South Africa, where black people African people constitute the largest group in all other provinces (Statistics South Africa 2012:21).

### Findings of the study

In examining the lived experiences of nurses concerning the family’s role in EOL care, three core themes emerged: the emotional challenges associated with the families of EOL patients, strategies that assist families and impediments nurses experience in providing the required support to families.

In Table 4, the themes, with their categories and sub-categories, are presented and then discussed in terms of related literature where applicable. Participant codes are indicated in Table 2 and Table 3.

**TABLE 2:** Demographic details of the professional nurse participants (P1–10).

Age (years)	Participant number	Gender	Ethnicity	Years of experience	Working unit
> 60	(P1)	F	MR	> 20	Medical
46	(P2)	F	B	2–5	Oncology
52	(P3)	F	MR	> 20	Medical
53	(P4)	F	MR	> 20	Oncology
57	(P5)	F	B	11–15	Medical
56	(P6)	F	MR	> 20	Medical
47	(P7)	F	MR	> 20	Medical
45	(P8)	F	MR	> 20	Oncology
46	(P9)	F	MR	4	Oncology
47	(P10)	F	MR	11–15	Medical

F, Female; MR, mixed race people; B, black people.

**TABLE 3:** Demographic details of Focus Group (F) 1 and Focus Group 2 (F2).

Participant number	Age (years)	Gender	Ethnicity	Years of experience	Unit
**Focus Group 1**					
Participant F1	51–60	F	MR	> 20	Oncology
Participant F2	51–60	F	MR	> 20	Medical
Participant F3	41–50	F	B	2–5	Medical
Participant F4	41–50	F	B	6–10	Medical
Participant F5	31–40	F	MR	6–10	Medical
Participant F6	20–30	M	B	2–5	Medical
**Focus Group 2**					
Participant F2(1)	41–50	F	MR	> 20	Medical
Participant F2(2)	31–40	F	B	2–5	Oncology
Participant F2(3)	31–40	F	MR	2–5	Medical
Participant F2(4)	31–40	F	B	2–5	Medical
Participant F2(5)	41–50	F	MR	6–10	Medical

F, Female; M, male; MR, mixed race people; B, black people.

**TABLE 4:** Results of the role of the family and EOL care.

Themes	Categories	Subcategories
1. Emotional challenges associated with the families of EOL patients	Supporting the patient and the family as a unit	-
	Emotional expressions	*Families’ grief and denial* *Displaced anger towards the staff* *Managing emotions* *Providing understanding and emotional support*
2. Strategies that assist families	Communication	*Communicating nursing procedures*
	Involving the family in the patient’s care	-
	Granting unfettered access	-
3. Impediments to providing the required care Need for specific training	Working environment is fast-paced, busy and understaffed	-
	Nurses’ need for emotional support	-
	Need for specific training	-

EOL, End-of-life.

## Theme 1: Emotional challenges associated with the families of end-of-life patients

Nurses recognise the emotional challenges patients and their families face in EOL care. They emphasise the importance of providing support and care to families, as they often experience trauma alongside the patient. Nurses understand the complex emotions that families may exhibit, such as grief, denial and anger towards healthcare staff. They also acknowledge the need to manage their emotions while navigating these diverse emotional responses. Nurses stress the importance of empathy and compassion in providing understanding and emotional support to families, even in challenging situations.

The analysis showed that EOL care requires a holistic approach from nurses in which the family is an important factor. Patients and their families are approached as a unit, requiring support from the participants. End-of-life situations are frequently associated with strong emotions expressed by the patient’s family and sometimes also experienced by the nurses themselves; however, they strive to provide understanding and support to the family regardless.

### Supporting end-of-life patients and their families as a unit

The patient and the family are a unit of interrelationships, and participants identify that both can be traumatised by the proximity of death; both need support from nurses, and in some instances, the family even more so:

‘[*I*]nvolve the family … because the cancer don’t have an impact only on the patient … but also on the family.’ (P9)

‘They need moral support, they need that care, especially the patient, especially the family.’ (F1)

‘[*Y*]ou must give more care to the family.’ (F4)

Martí-García et al. (2020:2) concur that patient and family suffer emotionally. Bloomer et al. (2021:548) conclude that prioritising the family’s needs should be at the core of EOL care.

### Emotional expressions

Steele and Davies (2015:51–72) state that patterns of behaviour and emotional expression of families in EOL situations can vary considerably. Participants experienced that families express emotions such as grief, denial and displaced anger, and nurses must often also contend with their emotional responses.

#### Families’ grief and denial

Peña-Vargas, Armaiz-Peña and Castro-Figueroa (2021:2) state that grief is a primary human emotion necessary for maintaining social bonds, and participants experienced that grieving families can express their grief in strongly emotional terms:

‘Because now when the patient’s family come, they are crying in the passage. Some people may be shouting or whatever they’re doing just to give, in Afrikaans, uiting aan hulle gevoelings [*expression to their feelings*].’ (P5)

Kübler-Ross (1974) identified five grief stages: denial, anger, bargaining, depression and acceptance. Just as patients pass through the stages of grief when given a terminal diagnosis, so too does their family, many of whom, participants found, are in denial about their loved ones’ diagnosis:

‘[*T*]he family of the palliative … they are in denial. They don’t want to accept the patient’s condition.’ (F4)

‘Because you can see even the members of the family, they will be really sad, they will be in denial, and they will be not accepting what is happening because they will be still having hope [*for*] their family member because they love them so much.’ (P3)

According to Tyrrell et al. (2023), denial is a defence mechanism against a distressing reality. They assert that a period of denial within the context of terminal illness is quite normal and an important part of processing difficult information.

#### Displaced anger towards the staff

Participants indicated that anger expressed by the family is often projected towards the nurses in the form of finding excessive fault with their work. Smeijers et al. (2021:122) express displaced anger as aggression towards ‘underserving others’:

‘But most of the time the family do have an attitude towards the staff, they find fault with everything you do, they find fault with the ward, this is not clean, stuff that’s got nothing to do with the patient, just because they’ve got this anger inside of them, the shock, they go through different emotions. So they take it out on us.’ (P4)

‘And sometimes you also see that anger and they want to take it on you because this is not right and this … And they always try to find fault. … And then we have to talk with our staff as well, just to explain to them that this is maar [*just*] how they’re gonna express their anger.’ (P6)

Tyrrell et al. (2023) confirm that anger is commonly experienced and expressed when the reality of a terminal illness becomes evident and is frequently directed towards medical providers. They advise that anger is a natural response, and understanding this response can help healthcare providers tolerate unpleasant, hurtful or unfounded accusations. They warn, however, that care should be taken to not just disregard criticism out of hand. Additionally, Gerber et al. (2020:6) note that sometimes, the families’ adversarial dealings with staff relieve the patients’ need to self-advocate and bridge a power imbalance between the patient and healthcare providers.

#### Managing emotions

Nursing terminal patients can be an emotional experience for nurses. Participants expressed having to deal with and manage the strong emotions expressed by patients and families while sometimes also needing to manage their own emotions:

‘And right then you have to be able to deal with all that emotions that the patient and the family is dealing with.’ (P10)

‘[*Y*]ou’re in the difficult position sometimes with the families and especially you’re also in shock because you also didn’t expect it to happen like that … and then there you stand, you must deal with your emotions and everything. It’s difficult.’ (F6)

‘To experience that emotion of the family also, it’s very difficult for us. Sometime[*s*] you can even certainly get emotionally involved.’ (F1)

#### Providing understanding and emotional support

The participants indicated that regardless of how families treat them or how they are feeling, they must address the family’s emotional needs and support them:

‘The more they complain, the more you show them compassion. So, go in there when they’re there, speak to them frankly. If they’ve got complaints solve it as soon as possible. Try to calm them down. Tell them about what you went through and how … maybe that can help them to go through their emotions. So, don’t alienate them at all.’ (P4)

‘[*S*]ometimes they feel sad if the patient is now not so better then, and they start crying and what must we do? We must give them a hug and tell them everything is gonna be fine. You must be there for the patient’s family as well and work towards the mutual goals.’ (F5)

Tyrrell et al. (2023) agree that nursing staff can empathise with the patient and the family and provide counsel, comfort and emotional support.

## Theme 2: Strategies that assist families

Nurses highlight several strategies that can assist families in EOL care. Effective communication is key, with nurses emphasising the need for transparent and accessible information delivery. Involving families in the patient’s care helps alleviate their distress and allows them to participate in caregiving actively. Granting families unfettered access, such as placing patients in single rooms and allowing overnight stays, creates a less traumatic experience of death and enables families to share meaningful moments. Nurses also emphasise the importance of clearly communicating nursing procedures to families, ensuring they understand the interventions being administered.

Participants indicated the strategies they employ to assist the family in coping with their loved one’s EOL situation. These included providing good communication, involving families in patient care and granting unfettered access to their loved ones when death is imminent.

### Communication

Most participants experienced the importance of good communication with the family. They stressed that the family must be fully informed in a language they can understand so they know what to expect:

‘So, the family is then fully informed and they know what to expect.’ (P9)

‘[*S*]peak the language that they will understand, not using the big terms.’ (P2)

The family is brought in for a meeting, and the doctor apprises them of the diagnosis and prognosis. A participant (P2) indicated that the sister must ensure the family has correctly understood the doctor and then give further explanation if required. Open communication is maintained with the family throughout the process, and their questions are answered:

‘[*C*]all in the family, we will have a meeting with the family and the patient.’ (P1)

‘[*W*]hen the doctors are speaking with the family I should be present in that conversation so that I know what the doctor has said to the family, up to what level, and then pick up…because sometimes they will use big terms. Then when doctor is gone, then I will ask them: do you guys understand? Yes, sister, I did understand. Repeat what he said and then I will pick up from there.’ (P2)

‘[*A*]nswer their questions, don’t ignore them, because that make it more difficult and communicate with the family constantly.’ (P4)

Gerber et al. (2020:2) argue that open and effective communication among patients, families and healthcare services is essential to EOL care. Nielsen et al. (2017) advocate increasing preparedness through interventions such as facilitating communication in the family about dying and ensuring that prognostic information is adapted to the family’s needs.

Steele and Davies (2015:51–72) confirm the need for families to be fully informed and that they need to be able to ask questions and have their questions answered in terms they can comprehend. They state that open and honest communication with nurses and the healthcare team is frequently the most important need of families and recommend communicating in straightforward terms about the patient’s condition and what the family can expect. Martí-García et al. (2020:e10516) assert that the experience of death was described by relatives as less traumatic when they received adequate information.

#### Communicating nursing procedures administered to the patient

The family becomes concerned for the patient when they do not understand why certain interventions are occurring. Participants gave examples of how it is important to communicate and explain the ‘what and why’ specific interventions are being done:

‘[*L*]et’s say the patient is not able to eat, they wouldn’t understand that the patient is not eating, then what are you doing here? And then you will explain there is a drip running at least to prevent the patient not to be dehydrated … So, you have to try and explain to them.’ (P2)

‘So, I told the family that is why we restrained him because he was pulling the catheter. So, the family member didn’t feel nice about it at all. So, I explained to him … we can’t just take it out now because the patient can even bleed mos now if you just take the catheter out. So, then they did understand.’ (F2)

### Involving the family in the patient’s care

Watching the deterioration of their loved one as a helpless onlooker can be distressing for family members. Some participants overcome this by involving the family in the patient’s care:

‘I try to be very, very, very friendly with the patient’s family so that I can involve them the whole time.’ (F5)

Participants expressed how they attempted to involve close family members in the patient’s care either in the hospital or to prepare them in the event they are sent home:

‘And I said to the visitor … will it be possible from tomorrow on to help and assist and to wash your mom because we’re short of staff and if it’s possible for you just to assist and help it will also make it so much eas[*ier*].’ (P9)

‘Communicate with them constantly. You can even ask them, if they’re around the bed and you, [*do you*] wanna turn the patient or change the linen or do something small, if they want to help you, just to get them involved in the care of the patient, especially if the patient is gonna be sent home.’ (P4)

This approach is confirmed by Steele and Davies (2015:60), who advise nurses to involve families in all aspects of care and encourage active participation in the patients’ physical care. This is especially important for children helping to care for a family member, as this helps them cope better with their grief. Similarly, Pun et al. (2022) observe that the more family members engage in the patient’s EOL journey, the less they experience psychological symptoms such as anxiety and depression after the patient’s death.

#### Granting unfettered access

Participants described caring for dying patients in a single room, when possible, so their families can have open access to them outside visiting hours. This included overnight stays for some family members to allow them to share the precious last moments with their loved ones:

‘We usually put them in a single room so the family can have access to that patient whenever they want to … That’s why we put them in a single room.’ (P4)

‘[*W*]e grant at least one or two permission to overnight and that’s where we offer them the lazy chairs to sit in, to be with their family member or their loved one … Because sometimes it is really a[*n*] honour for them to be with them, especially with their last words or last saying of that patient or what they can actually share with each other.’ (P9)

Martí-García et al. (2020) concur that family experiences of death are less traumatic when they can accompany family members in their final moments satisfactorily.

## Theme 3: Impediments to care

Nurses face both systemic and individual challenges in providing optimal EOL care. They express the strain of working in fast-paced, understaffed environments and call for systemic improvements to facilitate better care. Nurses also recognise their need for emotional support, as providing EOL care can affect their well-being. They emphasise the importance of specific training to equip healthcare providers with the skills required for comprehensive EOL care, highlighting the need for continuous education in this specialised field. This theme emphasises the multifaceted challenges that hinder optimal EOL care provision and calls for systemic and individual interventions.

The impediments in caring for patients and families relate to nurses’ busy schedules and understaffing issues. Further, dealing with EOL patients and families is very stressful, and without sufficient emotional support for themselves, nurses may lack the inner resources to provide optimal care. They further identified a need for specific training in dealing with and supporting families.

### The hospital setting is fast-paced, busy and understaffed

Participants identified challenges endemic to nursing in a tertiary hospital, such as being fast-paced, busy and typically understaffed, which hamper nurses’ ability to spend the requisite time and provide the support families may need:

‘[*A*] hospital setting, we are so busy.’ (P1)

‘Because in our setting it’s so fast and everything happens so fast that by the time that you wanted to go and talk to that tannie [*aunty*] or that oompie [*uncle*] then you don’t have the chance to even do that.’ (P5)

‘We try to give the best care we can but sometimes it isn’t possible with the limited staff that we have because they need more attention, more care, to be given longer periods that you need to attend to the patient and the family, because you need to explain everything to them.’ (P8)

As Level 3 academic hospitals, tertiary hospitals provide most of the specialist services in internal medicine, such as paediatrics, obstetrics, gynaecology and general surgery (Department of Health 2012). Lasater et al. (2019:302) observe that despite nurses’ proximity to EOL patients and their clinical abilities, they frequently remain an untapped resource because of the constraints and demands of their practice environment.

According to the Western Cape government (n.d.b), in-hospital palliative care is offered in tertiary hospitals to assist patients and communicate with their families, but this only sometimes translates into practice as nursing staff must attend to patients with a wide range of conditions. Additionally, there is an estimated shortage of between 26 000 and 62 000 professional nurses in South Africa, with an anticipated increase in demand between 305 000 and 340 000, by 2030 (RHAP n.d.).

### Nurses’ need for emotional support

According to Kostka, Borodzicz and Krzemińska (2021:706), taking care of dying patients and supporting their families is viewed as one of the most challenging clinical experiences for nurses and one of the leading causes of occupational burnout and compassion fatigue among nurses. Most participants expressed a need for emotional support to help them deal with the stresses of EOL care:

‘So, I strongly felt that we need to support the staff members in the unit … if they are at ease with their emotions then they can take [*care*] of our patients.’ (P9)

‘We are also human beings. To experience that thing every day is not nice. Maybe they can send us through counselling or something else.’ (F1)

Unaddressed psychological stressors experienced by nurses ultimately impact the quality of patient care (Hussain 2021:485), which extends to the care of the family. Boyle (2011) recommends support in the form of on-site counselling that is visible and accessible and offers practical solutions. She also suggests peer support groups, debriefing sessions and supportive workshops. Hussain (2021:485) recommends on-the-spot debriefing sessions and introducing programmes that promote self-care and reflective practices.

Povedano-Jiménez et al. (2021:9515) recommend implementing formal prevention and behavioural and emotional support programmes for nurses to assist them in preventing burnout.

### Need for specific training

In addition to palliative care training in general, nurses experienced a need for specific training on how to support, speak to and advise the family:

‘Training … more the psychological part as how you can support the family.’ (P4)

‘How do you speak to the family? What do you say to the family?’ (P7)

‘[*W*]hat do you tell the family [*on*] how you [*the family should*] treat the patient, how you cope with emotions of the patient, how you cope with your own emotion as a family member? … So, I think training in that regard will also be better.’ (P1)

According to Rosa et al. (2021:135), urgent worldwide curricular integration of palliative care is required to equip the global nursing workforce and deliver holistic, patient-centred care throughout the care continuum. The nurses’ EOL and palliative care duties must be revised to ensure the integration of high-quality, relationship- and value-centred care for patients and their families. The authors further assert that considering the recent COVID-19 epidemic and possible future public health crises, nurses at all levels of schooling must receive palliative care training.

The WHO (2014:6) urged member states to incorporate palliative care as a fundamental element of the continuous education and training provided to care providers, aligning with their specific duties and responsibilities. This endeavour is guided by principles such as that intermediate training should be available to all healthcare workers who regularly interact with patients facing life-threatening illnesses. This includes oncology, infectious diseases, paediatrics, geriatrics and internal medicine professionals.

## Limitations and strengths of the study

This study was conducted in a tertiary hospital, in an urban environment and a specific province in the Western Cape. The sample’s demographic profile primarily reflected women’s perspectives, as only one male participant existed. This could influence the transferability of the findings to other healthcare settings or hospitals in other provinces. These limitations are, however, mitigated to some extent by many of this study’s findings reflecting or concurring with other studies in different, diverse settings, which strengthens their credibility.

## Recommendations

Future studies could look at EOL care provision in different healthcare facilities and provinces of South Africa. Studies on how families perceive the support they receive or what they require from healthcare providers can be conducted. As South Africa is culturally diverse, the perspectives and needs of families and patients from different cultures should also be researched.

## Conclusion

This study explored and described the role of the family in EOL care. Distraught families in EOL situations can be viewed as metaphorical ‘patients’ who, together with their dying loved one, require nurses’ care, understanding and emotional support, often under challenging conditions.

Participants experience that open, ongoing and clear communication with the family during the EOL process, involving the family in the patient’s care and allowing them access to the patient’s last moments, assists families in coping with the painful experience.

The ability of nurses to provide families with the optimal care they need is negatively impacted by the fast-paced, busy and understaffed environment of a tertiary hospital. Therefore, nurses require emotional and training resources to address EOL patients’ and their families’ needs optimally.

The challenges nurses face could be addressed through continuous in-service training workshops that include training in communication skills. Counselling and reflective practice can assist healthcare workers in dealing with difficult situations when dealing with patient and their families. The emotional effect of EOL situations calls for the provision of psychosocial interventions.
